# Matrix Effect Evaluation in GC/MS-MS Analysis of Multiple Pesticide Residues in Selected Food Matrices

**DOI:** 10.3390/foods12213991

**Published:** 2023-10-31

**Authors:** Mateja Bulaić Nevistić, Marija Kovač Tomas

**Affiliations:** 1 Inspecto Ltd., Industrijska Zona Nemetin, Vukovarska Cesta 239b, 31000 Osijek, Croatia; mateja.bulaic@inspecto.hr; 2 Department of Food Technology, University North, Trg dr. Žarka Dolinara 1, 48000 Koprivnica, Croatia

**Keywords:** co-extractives effect, GC-MS/MS, pesticides, foods of plant origin, QuEChERS, matrix-matched calibration

## Abstract

Multi-analyte methods based on QuEChERS sample preparation and chromatography/mass spectrometry determination are indispensable in monitoring pesticide residues in the feed and food chain. QuEChERS method, even though perceived as convenient and generic, can contribute to sample matrix constituents’ introduction to the measuring system and possibly affect analytical results. In this study, matrix effects (ME) were investigated in four food matrices of plant origin (apples, grapes, spelt kernels, and sunflower seeds) during GC-MS/MS analysis of >200 pesticide residues using QuEChERS sample preparation. Data analysis revealed considerable analyte signal enhancement and suppression: strong enhancement was observed for the majority of analytes in two matrices within the commodity groups with high water content—apples, and high acid and water content—grapes (73.9% ME_S_ and 72.5% ME_A_, and 77.7% ME_S_ and 74.9% ME_A_, respectively), while strong suppression was observed for matrices within the commodity groups with high starch/protein content and low water and fat content—spelt kernels, and high oil content and very low water content—sunflower seeds (82.1% ME_S_ and 82.6% ME_A_, and 65.2% ME_S_ and 70.0% ME_A_, respectively). Although strong matrix effects were the most common for all investigated matrices, the use of matrix-matched calibration for each sample type enabled satisfactory method performance, i.e., recoveries for the majority of analytes (up to roughly 90%, depending on the fortification level and matrix type), which was also externally confirmed through participation in proficiency testing schemes for relevant food commodity groups with the achieved z-scores within acceptable range ≤ |2|.

## 1. Introduction

Agriculture production is nowadays unimaginable without the use of pesticides, protecting the crops from diseases and pest attacks and raising the yields and food quality, especially when the climate change scenarios are taken into account [1,2]. At the same time, monitoring their residues and exposure to those residues in the feed and food chain is essential and required to enforce legislation, but also to guarantee food safety [3]. Namely, once applied, pesticides undergo various processes, such as degradation to new substances, depending on its properties and environmental factors, as well as transfer from target to non-target organisms or area by adsorption, leaching, volatilization, spray drift, or run-off, thus impairing air, soil, and water, and consequently food safety [1,4,5]. Therefore, within the European Union (EU), maximum residue levels (MRLs) of pesticides in or on certain food and feed of plant and animal origin are set by Regulation (EC) No 396/2005 [6], and the EU Pesticides Database (https://food.ec.europa.eu/plants/pesticides/eu-pesticides-database_en, accessed on 18 July 2023) allows search for information on active substances used in plant protection products, MRLs in food products, and emergency authorizations of plant protection products in Member States. In the Republic of Croatia, the information on registered plant protective products is publicly available at the official website of the Ministry of Agriculture (https://fis.mps.hr/fis/javna-trazilica-szb/), accessed on 27 October 2023), where information such as trade names, permitted place of sale, distributor, product function, usage area, etc. can be found, facilitating the user’s proper pesticide application.

Considering the large number of pesticide formulations available and, therefore, the large number of residues possible, there is a need for multi-analyte methods capable of unambiguously determining multiple compounds at the same time. Above all, in order to properly prove food safety, it is important to ensure the reliability of the obtained testing results by using the validated method, whose performance characteristics were proven to correspond to the legislatively established quality criteria, especially for the needs of residue monitoring, often present at trace levels. Methods employing gas (GC) and liquid chromatography (LC) (depending on the properties of the analyte) for separation and mass spectrometry (MS) for identification/quantification purposes are thus imperative, as being more selective and sensitive compared to those with conventional detectors, enabling the use of simple sample preparation procedures, increasing throughput, saving time and money [7]. Except for the GC and LC, different techniques for determining pesticide residues in actual samples have also been utilized, such as capillary electrophoresis (CE) and enzyme-linked immunosorbent assay (ELISA), with limited applicability [8,9,10].

As numerous physically and chemically diverse substances must often be assessed instead of just one or a specific class of analytes, the development of sample preparation procedures for determining pesticide multi-residues in food samples is vital [8]. The Quick, Easy, Cheap, Effective, Rugged, and Safe (QuEChERS) sample preparation approach, initially developed by Anastassiades et al. [11], consisting of acetonitrile extraction and salt mixture partitioning followed by clean-up using dispersive solid phase extraction, is frequently employed for multi-residue analysis of pesticides in agricultural products. Although convenient, QuEChERS dispersive solid phase extraction (dSPE) is considered a soft purification technique, as removing only a small portion of the matrix from sample extract, possibly contributing to the phenomenon called the matrix effect, often causing inaccurate quantitation, especially being pronounced in GC-MS analysis [12].

Aside from QuEChERS, there is a number of other sample preparation strategies established, including solid-phase extraction (SPE), solid-phase microextraction (SPME), microwave-assisted solvent extraction, supercritical fluid extraction, or liquid–liquid extraction (LLE) [8,13], all of which have their role in pesticide residues determination, and are chosen depending on the target analyte characteristics, matrix type and analytical method quality requirements. In addition, more sample preparation technologies are still to be adopted for more accessible, affordable, and eco-friendly analysis to enable more rapid and efficient pesticide residue testing in food matrices [8].

Furthermore, it should be noted that more complex sample preparation methods and extensive extract clean-up often result in the loss of some analytes and increased labor and cost demands. Inadequate clean-up can, therefore, lead to adverse effects related to the quality of acquired data, such as masking of residue peaks by co-eluted matrix components, the occurrence of false positives, and inaccurate quantitation [14]. For instance, the classical sorbents used in the SPE method (e.g., C8, C18) may retain the analytes because of non-selective hydrophobic reactions, leading to the joint extraction of interfering substances and low matrix cleaning efficiency [13].

**Figure 1:**
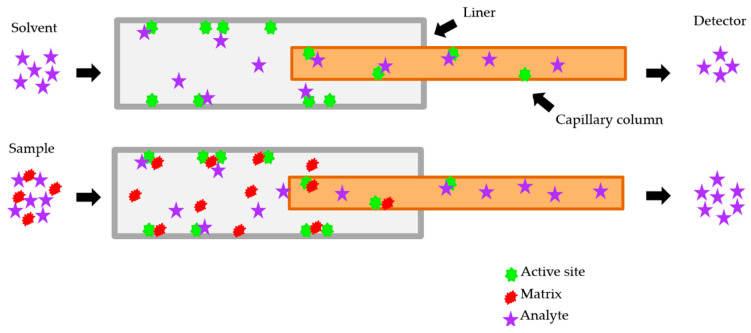
Illustration of the matrix-induced enhancement effect cause, modified from [16].

Regarding the matrix effects in GC–MS analysis, co-extracted matrix components often increase the response, resulting in analytes’ concentration overestimation. As shown in Figure 1, analytes injected into a GC interact with the column coating material and other surfaces, causing undesirable peak tailing and certain degradative effects, and thus, integration problems and impaired detectability. Most problematic interactions occur in the injector area, i.e., liner and column entrance, especially in the case of freshly cut columns. The exposed surfaces are covered with a film of non-volatile compounds originating from previous injections, increasing the activity. Certain molecules in the injected solution, such as matrix components, can mask the active sites, reducing those undesired outcomes and improving the analyte introduction into the column. This effect is known as the matrix-induced signal enhancement effect [11,15].

Matrix-induced enhancement (overestimation) was first studied and reported in detail by Erney et al., who investigated the GC determination of organophosphorus pesticides in fatty foods and proposed the mechanism of the occurring matrix effects. It was described that the matrix protects the analytes from adsorption or alteration, and this protection is not permanent and probably is dependent on the matrix’s nature and concentration [17,18]. To achieve acceptable results, the use of the matrix-modified standards prepared from the residue-free matrix of the same kind for calibration was proposed as a possible solution [18].

In general, the matrix effect depends on the physicochemical properties of the analytes, matrix type, and analyte/matrix ratio. The matrix effect can be compensated using three types of methods: eliminating matrix components or active sites, modifying the GC injection technique, and masking the active sites. However, not all of them are completely feasible in routine laboratory work. For instance, additional sample clean-up to eliminate matrix often results in low analyte recovery and higher overall analysis cost, similar to the use of internal standards. Keeping the GC system in an inactivated state is difficult, regardless of the frequent maintenance. GC injection techniques, aiming to shorten the time of analyte-active site contact, have limited effect, as they cannot directly act on active sites. The best choice seems to be the masking of the active sites in the GC system, using methods such as the addition of analyte protectants or matrix-matched calibration [11,12,15,19]. The latter is also recommended by the EU [20] as a matrix effect compensation method for residue measurements.

Although not obligatory to evaluate according to the Commission Decision (EC) No. 657/2002 concerning the performance of analytical methods [21], the SANTE guidance document on Analytical quality control and method validation procedures for pesticide residues analysis in food and feed suggests that matrix effect should be assessed at the initial method validation stage [20]. Accordingly, the aim of this study was to evaluate the effect of co-extractives of four different food matrices during the multi-residue analysis of >200 pesticides using QuEChERS sample preparation and gas chromatography coupled to tandem mass spectrometry (GC-MS/MS) and investigate the matrix-matched calibration as a convenient solution for the matrix effect compensation to achieve satisfactory method performance in routine laboratory work for food safety assessment.

## 2. Materials and Methods

*Chemicals*. Reference standards of pesticide residues were purchased from CPAchem (Bogomilovo, Bulgaria). All standard solutions were stored according to the manufacturer’s instructions and brought to room temperature before use. HPLC grade acetonitrile was supplied by KEFO d.o.o. (Sisak, Croatia), and ultrapure water was generated by the Niro VV system (Nirosta d.o.o., Osijek, Croatia). QuEChERS buffer-salt mixture packets (1 g trisodium citrate dihydrate, 1 g sodium chloride, 0.5 g disodium hydrogen citrate sesquihydrate, and 4 g of anhydrous magnesium sulfate) and dSPE salt mixtures (900 mg of anhydrous magnesium sulfate, 150 mg of primary secondary amine, and 150 mg graphitized carbon black or 150 mg C18E sorbent) were obtained from Phenomenex (Torrance, CA, USA).

*Sample preparation and spiking*. Food samples, including apples, grapes, spelt kernels, and sunflower seeds, belonging to four common commodity groups (Table 1) were obtained from retail stores and prepared for the analysis of pesticide residues (listed in Table A1, Appendix A) according to the standardized QuEChERS method (EN 15662). Homogenized samples were accurately weighed (10 g for fruits/vegetables, and 5 g for cereals/oilseeds) into plastic centrifuge tubes, extracted 1 min by shaking using 10 mL of acetonitrile, followed by the second 1 min extraction using QuEChERS buffer-salt mixture. After weighing, 10 mL of ultrapure water was added to the sample for cereals and oilseeds. After 5 min centrifugation at room temperature using Restek Q-sep 3000 centrifuge (Restek, Bellefonte, PA, USA), an aliquot of the acetonitrile phase was cleaned using dSPE salt mixture (for sunflower sample dSPE containing C18E) by shaking for 0.5 min, centrifuged and filtered through 0.22 µm nylon filter, afterward transferred to a glass vial. For the spiking experiment, the food samples were fortified at two levels with the appropriate amount of the analytical standard, mixed and left to equilibrate, and subsequently prepared according to the above-described QuEChERS procedure. Proficiency testing (PT) food samples used for external method confirmation were obtained from EU reference laboratories for pesticide residues (Almería, Spain and Kongens Lyngby, Denmark) and Bipea (Paris, France).

*GC-MS/MS analysis*. Instrumental determination of pesticide residues was performed using Trace 1300 gas chromatograph coupled to TSQ 8000 Evo tandem mass spectrometer (Thermo Scientific, Waltham, MA, USA) under the instrumental conditions previously described by Kovač et al. [22]. TraceFinder software (v. 3.3, Thermo Scientific, Waltham, MA, USA) was used for instrument control, data acquisition, and processing.

*Calibration and matrix effect evaluation*. A multi-residue standard solution of pesticides was prepared by mixing appropriate volumes of each pesticide standard mix and acetonitrile to obtain a 10 mg/mL solution, afterward diluted with pure acetonitrile or blank sample matrix to obtain five different working solutions (calibrants) in concentrations between 2.5 ng/mL and 100 ng/mL, corresponding to the analyte concentrations in the sample of 5 ng/g, 10 ng/g, 20 ng/g, 50 ng/g and 100 ng/g. Sample extracts used for standard solutions preparation were analyzed in advance to ensure they were pesticide residue-free.

The influence of co-extractives (i.e., matrix effect, ME) from the samples on the pesticide residue concentration measurements was calculated by comparing the slopes of matrix-matched calibration curves to solvent curves, according to the following equation:

(1) $${ME}_{S}\%=\left(\frac{{Slope}_{matrix-matched\;calibration\;curve}}{{Slope}_{solvent\;calibration\;curve}}-1\right)\times 100$$

where ME_S_ represents the matrix effect calculated using calibration curve slope.

For better understanding, the matrix effect was also estimated by the difference of detector response from pesticide residue standard in sample matrix extract (matrix-matched standard) and standard in pure solvent (acetonitrile) at the same concentration, as suggested by SANTE [20], according to the equation:

(2) $${ME}_{A}\%=\left(\frac{{Area}_{standard\;in\;matrix}}{{Area}_{standard\;in\;solvent}}-1\right)\times 100$$

where ME_A_ represents the matrix effect calculated using analyte response (area).

*Data analysis*. The obtained data for certain method performance characteristics were evaluated using Microsoft Excel 2016 (Microsoft, Redmond, WA, USA). Statistical data analysis was performed using IBM SPSS Statistics software, version 29.0.1.0.(171) (IBM Corp., Armonk, NY, USA). The evaluated dataset was composed of excluding outliers for each food matrix.

## 3. Results and Discussion

Four food matrices belonging to the four most common commodity groups were chosen to investigate the co-extractive effect during the GC-MS/MS multi-residue pesticide analysis. The obtained data on matrix effects were compared and evaluated according to the sample matrix type and elution time from the capillary column (retention time). According to the SANTE guidance document [20], in case of more than 20% signal suppression or enhancement, matrix effects need to be addressed. A value of 100% is therefore considered as no effect, ±20% values were considered as soft ME, ±50% values as moderate ME, and outside ± 50% values as strong ME, as previously suggested by Sulyok et al. [23,24] and Rutkowska et al. [24].

For a better understanding of the co-extractives effect, ME was estimated using both slope data (Equation (1)) as authors such as Kim et al. [25] or Shendy et al. [26], and area data (Equation (2)) as suggested by SANTE guidance document [20] or Rutkowska et al. [24]. In the latter area equation, the response of the targeted limit of quantification (LOQ) was chosen as a method threshold at which the interferences are considered to be the strongest. Although statistically significant differences could not be established for investigated matrices due to the nature of the dataset, certain analyte discrepancies were observed in the obtained slope (MEs) and area (ME_A_) values. For 19.8% of analytes belonging to various pesticide groups but dominantly organophosphates, ME variance in slope and area values was observed in at least one matrix type. In the case where these two values differ, area equation data should be considered since it is estimated at the mentioned method threshold value of LOQ, giving more accurate information on the real extent of the co-extractive effect [7].

**Figure 2:**
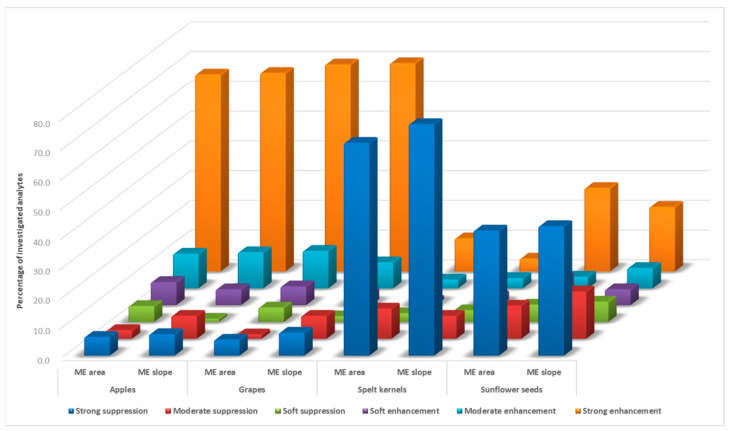
Summary of matrix effect (ME) values obtained using slope and area equations for analytes in investigated food matrices.

As presented in Figure 2, the strong matrix effect, both enhancement and suppression, was the most common for the investigated food matrices. For two matrices within the commodity groups with high water content—apples and high acid and water content—grapes, strong enhancement was recorded for the majority of analytes (73.9% ME_S_ and 72.5% ME_A_, and 77.7% ME_S_ and 74.9% ME_A_, respectively). On the other hand, for matrices within the commodity groups with high starch/protein content and low water and fat content—spelt kernels and high oil content and very low water content—sunflower seeds, strong suppression was recorded for the majority of analytes (82.1% ME_S_ and 82.6% ME_A_, and 65.2% ME_S_ and 70.0% ME_A_, respectively). With a strong co-extractive effect observed for the highest percentage of analytes among investigated food matrices, spelt kernels proved to be the most complex matrix. Nevertheless, signal suppression in the latter two matrices is unlikely the result of the matrix effect in the narrow sense described above, but probably the consequence of the large matrix peaks co-eluting with the analytes, interfering with the ionization and therefore reducing the signal intensity of the fragments in the MS.

**Figure 3:**
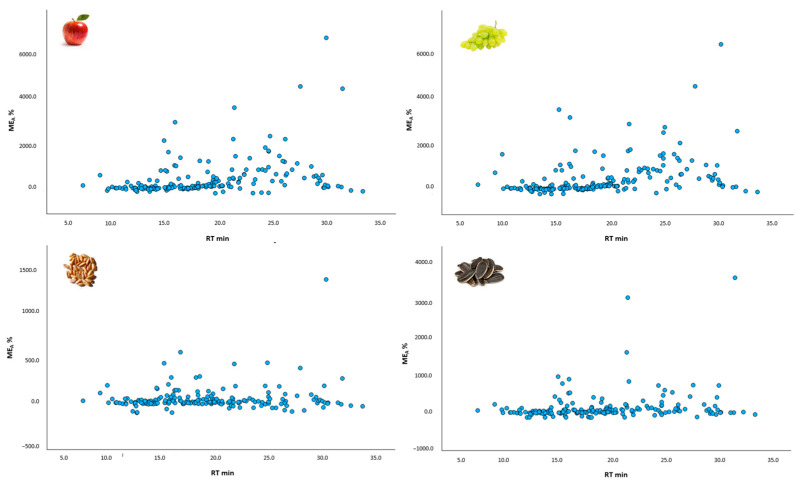
Correlation of matrix effect (ME_A_) in investigated food matrices (apples, grapes, spelt kernels, and sunflower seeds) with retention time (RT).

Even though the co-extractives effect is generally considered unpredictable, depending not only on matrix type but also on certain analytes, ME_A_ values were plotted against retention time to investigate possible patterns between matrix type and analytes’ characteristics that could be useful for future method extension in case of adding new compounds. For all four matrices, grouped ME_A_ data (Figure 3) can be observed at low RT (10–20 min) and more scattered data distribution at retention time > 20 min, especially being pronounced for two matrix groups with high water content—apples and grapes.

Pearson’s correlation was significant at the 0.01 level (2-tailed) for bivariate correlation between retention time and area value variables for apples, grapes, and sunflower seeds matrices, with correlation values of 0.357, 0.353, and 0.209, respectively. The spelt matrix showed no significant correlation values. Although statistically significant, correlation coefficient values close to 0 indicate no linear relationship between retention time and analyte area response.

**Figure 4:**
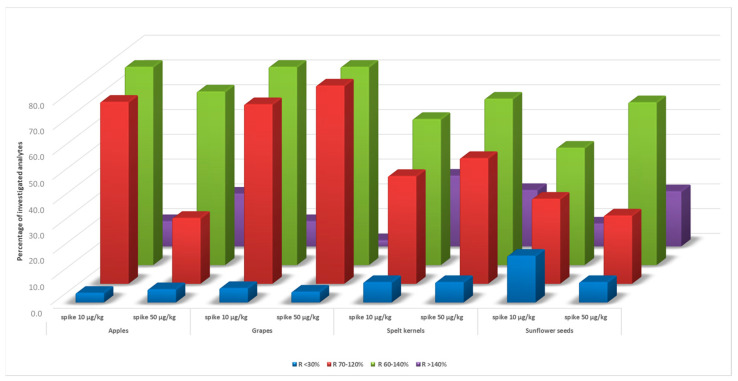
Summary of recoveries (R) obtained for analytes in investigated food matrices.

The obtained matrix effect values demonstrated the need for co-extractive effect compensation, which in this case was the use of matrix-matched calibration for each matrix type. In order to evaluate the matrix-matched calibration as a solution for the matrix effect compensation, spiking experiments were performed at two different fortification levels: at the low level of 10 µg/kg, the method LOQ, and at a high level of five times the LOQ value. The values within 60% and 140% were found acceptable, corresponding to the EU guidelines, i.e., the practical default range in the routine analysis set by the SANTE guidance document [20]. In general, acceptable recovery values were obtained for up to roughly 90% of the investigated compounds, depending on the fortification level and matrix type. As presented in Figure 4, the highest percentage of analytes within the acceptable recovery range was found for apples at the low fortification level of LOQ (82.1%) and for grapes at the high fortification level (88.9%), while the lowest for sunflower seeds at both levels (47.3% and 65.7%, respectively), which is in accordance with the ME data. In contrast, when using solvent calibration instead of matrix-matched, the percentage of analytes outside the specified acceptable recovery range (60–140%) was up to 85.8 for apples, 68.1 for grapes, 55.6 for spelt kernels, and 41.5 for sunflower seeds.

As matrix-matched calibration reduces the bias only for the matrix effects [20], a certain percentage of analytes outside the SANTE recovery acceptance threshold values—below 30% (3.9–18.8%) and above 140% (2.4–28.5%) was also observed. In such cases, assuming analyte peak shape and response are satisfactory, mathematical correction for recovery imposes as the solution to correct bias for both matrix effect and other losses, e.g., losses due to extraction and clean-up losses. Nevertheless, additional (sample) method optimization could address the abovementioned issue, but also the use of other approaches. Although standard addition or isotopically labeled internal standards are generally considered the most effective way to compensate for matrix effects [20,27], their use can be regarded as time-consuming and expensive, inadequate for most routine laboratory work. On the other hand, using analyte protectants such as ethylene glycol, added to both the sample extracts and the calibration standard solutions to equalize the response of compounds in solvent calibrants and sample extracts stands out as the method of choice [12,20]. As highlighted by Rahman et al., the combined use of analyte protectants and matrix-matched calibration could be the most appropriate solution to enable acceptable recoveries [19]; for instance, Čajka et al. performed in 44 pesticide residues determination, –achieving satisfactory results for the analyzed compounds [28]. However, analyte protectant application requires additional equipment for its continuous introduction into the carrier gas. At the same time, the necessity of establishing the optimum protectant for each analyte has also been pointed out [12], and the possible use of several protectants’ mixture, as in the case of Soliman et al., where seven protectants were optimized for the determination of 224 pesticides in the strawberry matrix [29].

Method performance was additionally proved through participation in available PT schemes for relevant food commodity groups, in which the achieved z-scores were within the desirable range of |z| ≤ 2, as presented in Table 2. Moreover, this GC-MS/MS method for pesticide residue analysis was already employed by Kovač et al. in the study of determining cereal’s contamination, i.e., safety and compliance with the legislative requirements, revealing cypermethrin and pirimiphos-methyl as significant insecticide residues in analyzed unprocessed cereal crops grown in Croatian fields [22].

Other authors also found the co-extractive effect to be the major obstacle in the quantitative trace-level analysis of pesticides, thus exploring various approaches for its minimization. Cho et al. investigated three calibration models, including matrix-matching and analyte protectants, for the multi-residue QuEChERS-based analysis of 113 residues in three food matrices (brown rice, black pepper, and mandarin orange) using GC-MS/MS. The slope equation was used for matrix effects calculation, which were at practical values (less than 30.0%) appropriate for routine analysis for most of the tested pesticides with all tested calibration options [30]. Kim et al. investigated 11 pyrethroid insecticides in animal-derived foods using GC-MSMS and modified the QuEChERS method, dealing with the matrix effect using matrix-matched calibration. Matrix effects, calculated by the slope approach, were in the range from −35.8% to 56.0% under the optimized clean-up conditions [25]. QuEChERS extraction followed by GC-MS/MS determination for 216 pesticide and metabolites determination in soil was explored by Łozowicka et al. Matrix effect values were obtained using a slope equation, and for most pesticides, signal enhancement was recorded, with a soft matrix effect observed for 87.0% of pesticides, moderate for 10.6%, and strong only for 2.4% of pesticides [31]. According to Xu et al., polarity and stability are the key contributors to analyte response alteration caused by matrix co-extractives. For instance, highly polar compounds, such as organophosphates, have the potential for high adsorption interaction with active sites and are susceptible to response alteration induced by the food matrix [32], which was also shown in our study, e.g., for the aforementioned insecticide pirimiphos-methyl, having the strong matrix effects (outside ± 50%) in all investigated food matrices, but satisfactory recoveries when using matrix-matched calibration for quantification. In addition, all authors also emphasized that the QuEChERS method is generic, requiring special attention during method development to minimize the sample co-extracts entering the measuring instrument and causing the matrix effect, thus affecting the analytical result.

## 4. Conclusions

Co-extractive effect evaluation was performed for four different food commodities—apples, grapes, spelt kernels, and sunflower seeds, during the multi-residue GC-MS/MS analysis of >200 pesticides using QuEChERS sample preparation. Both signal suppression and signal enhancement were observed for all four matrices, and their extent was dependent on the analyte/matrix combination. For high water content commodity—apples and high acid and water content commodity—grapes, strong signal enhancement was observed for the majority of analytes (73.9% ME_S_ and 72.5% ME_A_, and 77.7% ME_S_ and 74.9% ME_A_, respectively). In contrast, for high starch and/or protein content and low water and fat content—spelt kernels and high oil content and very low water content—sunflower seeds, signal suppression was the most common for the investigated analytes (82.1% ME_S_ and 82.6% ME_A_, and 65.2% ME_S_ and 70.0% ME_A_, respectively). Certain discrepancies were observed in the matrix effects calculated using area and slope equations; however, they both emphasized the need for co-extractive effect compensation. Although a strong co-extractive effect was observed as the most common for all investigated matrices, the use of matrix-matched calibration enabled satisfactory recoveries for the majority of analytes, which was also externally confirmed via successful participation in proficiency testing schemes for relevant food commodity groups. Nevertheless, gathered research results leave room for additional optimization to achieve even better method performance.

## Figures and Tables

**Table 1:** Investigated food commodity groups and food commodities classified according to the SANTE guidance document [20].

Commodity Group	Commodity Category within the Group	Representative Commodity within the Category
High water content	Pome fruit	Apples
High acid content and high water content	Small fruit and berries	Grapes
High starch and/or protein content and low water and fat content	Cereal grain and products thereof	Spelt kernels
High oil content and very low water content	Oil seeds	Sunflower seeds

**Table 2:** PT results for pesticide residues in relevant food commodity groups.

Commodity Group	PT Scheme (Matrix)	Analyte	z-Score
High water content	EUPT-FV23 (Aubergine)	Chlorfenapyr	0.1
		Diazinon	−0.5
		Endosulfan sulfate	0.6
	EUPT-FV24 (Tomato)	Chlorfenvinphos	−1.2
		Deltamethrin	1.5
		Diazinon	−0.4
		Fenamiphos	0.7
		Procymidone	−0.1
High acid content and high water content	BIPEA 19a-359 (Blackberry)	Lindane	−1.7
	EUPT-SRM12 (Strawberry)	Chlorothalonil	−1.8
	EUPT-FV19 (Lemon)	Chlorfenapyr	0.8
		Diazinon	0.6
		Ethoprophos	0.2
High starch and/or protein content and low water and fat content	EUPT-CF14 (Rice kernels)	Isoprothiolane	−0.1
		Profenofos	0.7
		p,p-DDE	0.2
	EUPT-CF16 (Barley kernels)	Endosulfan-beta	0.0
		Fenpropathrin	0.1
		Lambda-cyhalothrin	−0.6
		Lindane	0.6
High oil content and very low or intermediate water content	EUPT-CF15 (Rapeseed cake)	Aldrin	−1.8
		Tefluthrin	−1.2
	EUPT-FV-SC03 (Avocado)	Bromopropylate	0.4
		Chlorpropham	−0.0
		Cypermethrin (sum of isomers)	0.1
		Diazinon	0.4
		Orthophenylphenol	−0.2
		Permethrin (sum)	0.1
		Phosmet	−0.1
		Procymidone	0.0

## Data Availability

Data is contained within the article.

## Appendix A

**Table A1:** Parameters for GC–MS/MS determination of pesticide residues.

Analyte	Retention Time (min)	Precursor Ion (m/z)	Production Ion (m/z)	Collision Energy (V)	Precursor Ion (m/z)	Production Ion (m/z)	Collision Energy (V)	Precursor Ion (m/z)	Production Ion (m/z)	Collision Energy (V)
2,4-DDD	19.85	237.1	165.1	20	235.0	165.2	20			
2,4-DDE	18.35	248.0	176.2	30	246.1	176.2	30			
2,4′-DDT	20.95	236.8	165.1	20	235.0	165.1	20	235.0	199.0	15
4,4′-DDD	21.35	237.0	165.1	25	235.0	165.2	20	235.0	199.1	15
4,4′-DDE	19.55	318.0	248.0	20	246.0	176.1	30	317.8	246.0	20
4,4′-DDT	22.45	236.8	165.0	22	165.1	164.3	15	235.1	165.0	25
Acrinathrin	25.75	181.1	127.1	26	181.1	151.2	30	181.1	152.1	20
Aldrin	16.15	262.9	190.9	35	262.7	191.0	30	262.9	193.0	30
Atrazine	12.93	215.1	200.0	5	200.0	132.0	8	200.1	122.2	10
Atrazine-desethyl	11.90	172.0	69.1	15	172.0	94.1	15	172.0	104.1	15
Azinphos-ethyl	26.85	160.0	77.0	16	132.0	51.0	26	132.0	77.0	12
Azinphos-methyl	25.80	160.0	50.9	34	132.0	77.0	12	160.0	77.0	16
Benfluralin	11.75	292.1	160.1	20	276.1	202.1	15	292.1	264.0	10
Bifenox	24.95	341.1	281.0	12	172.9	137.9	16	341.0	189.0	20
Bifenthrin	23.65	181.2	165.2	25	181.0	179.0	12	181.2	166.2	10
Bioresmethrin	22.75	171.0	128.0	14	123.1	81.1	8	143.0	128.1	10
Bromfenvinphos	18.90	323.1	266.9	10	266.9	159.0	14	266.9	203.0	10
Bromfenvinphos-methyl	17.70	294.9	79.1	30	109.0	79.0	6	294.9	109.0	16
Bromophos-ethyl	18.05	302.7	284.8	14	96.9	65.0	16	96.9	78.9	12
Bromophos-methyl	16.80	331.0	316.0	15	328.9	313.9	20	330.8	315.8	14
Bromopropylate	23.95	340.8	185.0	14	184.9	75.5	30	184.9	156.9	12
Butachlor	18.35	237.0	160.0	5	160.0	131.7	12	176.1	147.0	12
Butamifos	18.83	286.0	185.0	24	200.0	65.1	20	286.0	202.0	14
Cadusafos	12.00	213.0	89.1	12	159.0	96.9	16	159.0	130.9	8
Carbophenothion	21.90	342.0	157.0	10	157.0	45.0	12	199.0	142.9	10
Chlorbenside	18.55	125.0	62.8	28	125.0	89.0	16	125.0	99.0	16
Chlorbufam	12.88	223.0	127.0	12	127.0	65.0	35	127.0	100.0	15
Chlordecone hydrate	21.45	271.7	140.9	36	271.7	234.8	16	271.7	236.8	12
Chlordene	13.75	66.1	39.1	20	66.1	40.1	15	66.1	65.1	10
Chlorfenapyr	20.35	248.9	112.0	24	136.9	102.0	12	248.9	137.1	18
Chlorfenprop-methyl	11.25	196.0	165.1	10	165.0	102.0	18	165.0	137.0	10
Chlorfenson	19.45	177.0	113.1	10	111.0	75.1	15	175.0	111.0	10
Chlorfenvinphos	17.55	323.0	266.9	14	266.9	159.0	16	266.9	203.0	10
Chlormephos	9.69	234.0	121.1	10	121.0	65.0	10	154.0	65.0	16
Chlorobenzilate	20.50	139.0	74.9	26	111.0	75.1	14	139.0	111.0	12
Chloroneb	10.33	206.0	190.9	12	190.9	113.0	14	190.9	141.0	10
Chlorothalonil	15.40	265.8	133.0	36	228.8	168.0	8	265.8	170.0	24
Chlorpropham	11.70	213.0	127.0	14	171.0	127.0	8	213.0	171.0	6
Chlorpyrifos-methyl	14.88	285.9	93.0	20	125.0	47.0	12	125.0	79.0	6
Chlorthal-dimethyl	16.13	300.7	222.9	22	222.7	166.9	20	300.7	272.9	12
Chlozolinate	17.25	331.0	259.0	8	259.0	152.9	26	259.0	187.9	12
cis-Chlordane	18.75	376.6	268.0	20	372.9	266.1	20	374.9	265.8	20
Coumaphos	28.25	362.0	109.0	15	226.0	163.0	18	362.0	226.0	15
Cyanazine	16.55	198.0	55.1	24	198.0	91.0	10	198.0	157.0	8
Cyanophenphos	22.10	169.0	77.1	22	157.0	77.1	22	169.0	141.0	8
Cyanophos	13.45	243.0	109.0	10	125.0	79.0	60	125.0	96.9	6
Cyflutrin beta 1	28.45	163.0	65.1	26	163.0	91.1	12	163.0	127.1	6
Cyflutrin beta 2	28.65	163.0	65.1	26	163.0	91.1	12	163.0	127.1	6
Cyflutrin gama 1	28.80	206.0	151.1	18	163.0	91.1	12	163.0	127.0	6
Cyflutrin gama 2	28.90	206.0	151.1	18	163.0	91.1	12	163.0	127.0	6
Cyhalofop butyl	25.90	256.0	91.1	24	256.0	120.0	10	256.0	157.8	30
Cyhalothrin gamma	25.75	208.1	151.8	28	181.0	151.9	22	208.1	180.9	8
Cyhalothrin lambda	25.35	208.1	180.9	8	180.9	151.9	22	197.0	141.1	10
Cypermethrin peak 1	29.05	180.9	152.1	20	163.0	91.1	12	163.0	127.1	6
Cypermethrin peak 2	29.25	180.9	151.9	18	163.0	91.1	12	163.0	127.0	6
Cypermethrin peak 3	29.35	163.0	91.0	12	163.0	127.0	6	163.0	152.1	12
Cypermethrin peak 4	29.45	180.9	152.2	20	163.0	91.1	12	163.0	127.1	6
Deltamethrin	31.98	252.8	92.9	16	181.0	152.1	22	252.8	172.0	8
Demeton O	11.15	171.1	115.0	10	88.1	59.8	6	89.1	61.0	8
Demeton S	12.48	170.0	114.0	8	114.0	81.0	14	142.5	114.9	6
Desmetryne	14.58	213.1	58.1	10	213.1	170.9	8	213.1	198.1	8
Diallate	12.18	234.1	150.0	20	86.1	43.1	5	234.1	192.1	10
Diazinon	13.13	179.1	121.5	26	137.1	54.1	20	137.1	84.1	12
Dichlofluanid	15.90	223.9	123.0	10	123.0	51.0	32	123.0	77.0	18
Dichlone	14.50	191.0	135.0	15	163.0	135.0	10	191.0	163.0	8
Dichlorbenzophenon p,p′	16.95	249.94	138.97	10	138.97	110.97	15	139.0	111.0	12
Dichlorvos	8.26	186.9	93.0	12	109.0	79.0	6	185.0	93.0	12
Diclofenthion	14.45	278.9	222.9	15	222.9	204.9	15			
Diclofop-methyl	22.65	340.0	253.0	10	252.9	126.9	36	253.0	162.1	15
Dicloran	13.28	205.9	147.9	20	175.9	148.0	10	205.9	176.0	10
Dicofol	16.98	250.0	139.0	10	111.0	75.0	15	139.0	111.0	15
Dieldrin	19.80	262.9	193.0	28	82.1	81.1	6	108.1	107.0	6
Dinitramine	13.68	260.7	194.7	18	215.9	196.0	8	260.7	241.0	8
Dioxabenzofos	12.18	216.0	138.0	8	183.0	153.0	8	216.0	201.0	8
Dioxathion	13.15	153.0	96.9	10	96.9	65.0	16	125.0	97.0	6
Diphenylamine	11.55	167.1	139.4	26	167.1	140.1	18	167.1	166.1	16
Disulfoton	13.60	185.9	96.9	16	88.0	45.0	18	88.0	59.8	6
Disulfoton sulfone	18.65	213.0	96.9	8	213.0	125.0	10	213.0	153.0	5
Disulfoton sulfoxide	8.78	213.0	96.9	18	125.0	97.0	6	153.0	97.0	10
Edifenphos	13.35	310.0	109.0	26	172.9	65.1	30	172.9	109.0	10
Endosulfan alpha	18.82	240.9	170.0	20	194.7	125.0	22	240.9	205.9	10
Endosulfan beta	21.5	240.9	205.9	15	158.9	123.0	12	194.7	159.0	8
Endosulfan sulfate	22.90	271.8	237.0	10	238.9	204.0	15	271.7	235.0	12
Endrin	20.50	280.0	245.0	8	245.0	173.0	22	262.0	192.0	30
Endrin aldehyde	21.95	344.9	281.0	8	173.0	138.1	16	249.8	214.9	24
EPN	24.2	169.0	77.0	22	157.0	77.0	22	169.0	141.0	8
EPTC	9.00	189.1	43.1	16	128.1	43.1	10	189.1	128.1	6
Esfenvalerate	31.08	181.1	152.1	20	125.0	89.0	22	167.0	125.0	12
Ethalfluralin	11.57	315.9	276.1	8	276.0	202.0	14	276.0	248.1	8
Ethion	20.75	230.9	128.9	22	153.0	97.0	10	230.9	174.9	12
Ethoprophos	11.50	200.0	158.0	6	157.9	96.9	16	157.9	113.9	6
Etofenprox	29.65	163.1	77.1	32	163.1	107.1	16	163.1	135.1	10
Fenamiphos	18.90	303.1	195.2	8	154.0	139.0	10	216.9	202.0	12
Fenamiphos sulfone	23.87	320.0	213.9	14	320.0	249.1	18	320.0	292.1	8
Fenamiphos sulfoxid	23.96	304.0	122.0	15	304.0	196.0	10	304.0	234.0	10
Fenchlorphos	15.20	287.0	272.0	20	124.9	79.0	6	285.0	267.0	13
Fenchlorphos oxon	14.30	304.0	109.0	18	269.0	109.0	15	304.0	269.0	15
Fenclorim	12.56	224.0	104.1	26	189.0	104.1	16	224.0	189.0	12
Fenitrothion	15.80	277.0	109.0	16	125.0	79.0	8	277.0	260.0	6
Fenpicoxamid	15.90	143.1	128.1	15	128.1	102.1	25	128.1	127.1	20
Fenpropathrin	24.10	181.0	126.8	28	97.1	55.1	6	181.0	151.9	22
Fenpropidin	15.10	98.2	41.5	18	98.2	55.1	14	98.2	70.0	10
Fenson	17.05	141.0	50.9	30	77.0	51.0	14	141.0	77.0	8
Fensulfothion	20.90	307.9	293.0	8	293.0	97.0	10	293.0	125.0	10
Fenthion	16.25	278.0	109.0	18	245.3	125.0	12	278.0	169.0	14
Fenthion oxon	15.25	262.1	247.1	10	109.0	79.1	5	247.0	77.1	30
Fenthion oxon sulfone	20.00	215.0	109.0	12	109.0	79.0	8			
Fenthion oxon sulfoxide	19.90	278.0	263.0	6	263.0	109.0	16			
Fenthion sulfone	21.09	310.0	109.0	24	310.0	125.0	16	310.0	136.0	18
Fenthion sulfoxide	20.93	125.0	79.0	8	109.0	79.0	8			
Fenvalerate	30.75	225.0	119.0	18	125.0	89.0	22	167.0	125.0	12
Flucythrinate	29.60	451.0	199.0	10	157.0	107.0	15	199.0	157.0	10
Fonofos	13.35	246.0	109.0	14	137.0	109.0	6	246.0	137.0	6
Formothion	14.61	170.0	93.0	8	125.0	79.0	8	126.1	93.0	6
HCH alpha	12.65	218.89	182.91	8	216.89	180.91	8	216.9	180.9	8
HCH beta	14.31	218.9	182.9	8	182.9	147.0	10	216.9	180.9	8
HCH delta	15.05	218.9	182.9	10	181.0	145.0	15			
HCH gamma	13.55	218.9	183.0	5	180.9	145.0	10	216.9	180.9	8
Heptachlor	15.20	273.8	238.8	15	100.0	65.1	10	271.8	236.9	15
Heptachlor epoxide	17.61	354.9	264.9	10	81.1	53.1	10	352.9	262.9	10
Heptenophos	10.80	215.0	200.0	8	124.0	62.9	28	124.0	89.0	12
Hexabromobenzene	24.49	551.6	391.7	34	231.8	151.0	24			
Hexachlorobenzene	12.88	285.8	213.9	30	283.8	213.9	30	283.8	248.9	20
Iodofenphos	19.05	376.8	361.8	16	125.0	47.0	12	125.0	79.0	6
Iprodione	24.18	316.0	247.0	15	314.0	245.0	15	314.0	271.0	10
Isodrin	17.15	194.9	159.0	20	66.1	65.1	10	192.9	123.0	30
Isofenphos	17.25	213.0	121.0	14	185.0	121.0	10	213.0	185.0	6
Isofenphos-methyl	16.85	241.1	121.1	20	199.0	65.0	34	199.0	121.0	10
Isoprotiolane	19.25	290.0	118.0	12	162.1	85.0	20	204.0	118.0	8
Leptophos	25.30	171.0	51.0	38	171.0	77.1	18	171.0	124.3	10
Malaoxon	14.65	127.0	99.0	6	99.0	71.0	8	127.0	109.0	12
Malathion	15.65	173.1	99.0	12	92.8	63.0	8	125.0	79.0	8
Mefenpyr-diethyl	23.30	299.0	253.1	10	253.0	127.7	36	253.0	189.3	22
Metaldehyde	6.55	208.0	176.0	5	89.0	45.0	10	117.0	45.0	10
Methacrifos	10.05	240.0	180.0	10	125.0	79.0	8	180.0	93.0	10
Methidathion	18.35	302.6	284.9	14	145.0	58.0	14	145.0	85.0	6
Methoxychlor	24.15	227.1	141.1	35	227.1	169.1	25	227.1	212.1	12
Mirex	26.00	273.8	238.9	15	236.8	142.9	20	271.8	236.9	15
Molinate	10.65	187.1	126.1	6	126.1	55.1	12	126.1	83.1	6
Nitrofen	20.80	283.0	162.1	20	202.0	139.0	21	283.0	252.9	10
Nitrothal-isopropyl	16.65	236.0	194.0	8	194.0	120.0	18	194.0	148.0	10
Ortho-phenylphenol	10.55	170.1	115.0	34	141.1	115.1	14	170.1	141.1	22
Oxadiazon	19.10	174.9	76.0	28	174.9	112.0	12	174.9	147.2	6
Oxychlordane	17.45	388.8	262.9	15	184.9	84.9	26	184.9	121.0	12
Oxyfluorfen	19.80	300.0	167.0	25	252.0	196.1	20	300.0	223.0	20
Paraoxon-ethyl	15.50	149.0	91.1	10	109.0	81.0	10	149.0	102.0	16
Paraoxon-methyl	14.05	230.0	105.9	16	95.9	65.0	12	109.0	79.0	6
Parathion-ethyl	16.53	291.0	109.0	12	109.0	81.0	10	124.9	97.0	6
Parathion-methyl	15.19	263.0	109.0	12	124.9	47.0	12	124.9	79.0	6
Pebulate	9.87	161.0	128.0	3	128.1	57.1	8			
Pentachloroaniline	14.75	264.8	193.9	20	262.9	191.9	20	264.8	229.9	10
Pentachloroanisole	12.91	279.9	143.0	38	236.9	119.0	18	264.9	236.9	12
Pentachlorobenzene	10.68	249.9	214.9	15	247.9	142.0	40	247.9	212.9	15
Pentanochlor	15.95	143.1	106.1	25	141.0	77.1	30	141.0	140.5	6
Penthiopyrad	20.78	152.1	80.1	18	152.1	124.1	8	152.1	134.1	8
Permethrin cis	27.25	183.1	153.0	12	163.0	91.1	12	183.1	168.0	12
Permethrin trans	27.55	183.0	153.0	14	183.0	165.1	10	183.0	168.1	10
Pethoxamid	17.65	260.0	147.0	15	131.0	91.0	10			
Phentoate	17.55	274.0	121.0	10	121.0	77.0	22	246.0	121.0	8
Phorate	12.15	260.0	75.0	8	75.0	47.0	8	121.0	65.0	8
Phosalone	25.75	182.0	74.8	30	121.1	65.0	10	182.0	111.0	14
Phosmet	24.40	160.0	50.9	38	160.0	76.9	22	160.0	133.0	10
Phosmet oxon	22.65	301.0	160.0	20	160.0	76.0	15	160.0	104.0	15
Phosphamidon	14.45	264.1	127.0	12	127.0	94.9	16	127.0	109.0	12
Pirimiphos-ethyl	16.4	318.1	166.1	12	304.0	168.1	12	318.1	182.1	10
Pirimiphos-methyl	15.35	305.1	180.1	8	290.1	125.0	20	290.1	233.0	8
Procymidone	17.93	283.0	96.1	10	96.0	53.0	16	96.1	67.1	10
Profenofos	19.25	336.9	266.9	12	296.7	268.9	10	336.9	308.9	8
Propanil	14.95	219.0	163.0	10	160.9	125.7	16	161.0	99.0	25
Propyzamide	13.35	172.9	74.0	38	172.9	109.0	26	172.9	145.0	14
Prothioconazole	24.50	232.1	53.1	18	116.1	89.1	10	232.1	116.1	10
Prothiofos	19.05	308.9	239.0	14	266.7	220.9	18	266.7	238.9	8
Pyrazophos	26.45	231.9	204.1	10	221.0	148.7	14	221.0	193.1	8
Pyridalyl	29.85	204.0	148.1	18	163.8	146.1	12	204.0	176.1	10
Quinalphos	17.73	157.1	102.0	22	146.0	118.1	10	157.1	129.0	14
Quintozene	13.58	248.8	213.9	15	236.8	119.0	25	236.8	142.9	25
Resmethrin	22.65	143.1	128.1	10	123.1	81.1	10	128.1	127.1	20
Simazine	12.95	186.0	91.0	8	172.7	138.0	6	172.7	172.2	8
Sulprofos	21.35	322.0	156.1	10	156.0	108.0	30	156.0	141.0	14
Tecnazene	11.55	214.8	143.6	20	214.8	178.7	10	214.8	179.9	15
Tefluthrin	13.55	197.0	141.1	10	177.0	127.0	14	177.0	137.0	16
Terbufos	13.05	230.9	128.9	22	230.9	174.9	12	230.9	203.0	8
Tetrachlorvinphos	18.48	330.8	109.0	18	109.0	79.0	6	328.9	109.0	18
Tetradifon	25.45	159.0	74.8	32	159.0	111.0	20	159.0	131.0	10
Tetramethrin	24.10	164.0	107.1	12	164.0	135.1	8			
Thiometon	12.58	125.0	47.0	14	88.0	59.8	6	125.0	79.0	8
Tolclofos-methyl	15.08	266.8	252.0	12	265.0	219.9	20	265.0	250.0	12
Tolfenpyrad	33.10	383.1	145.1	10	145.0	117.0	12	383.1	171.1	20
trans-Chlordane	18.6	374.7	265.9	22	271.7	236.8	12	373.0	264.1	20
Triallate	13.75	268.0	183.9	18	86.1	43.3	6	268.0	226.0	12
Trichloronate	16.65	297.0	269.0	12	268.9	222.9	20	270.8	224.9	22
Trifluralin	11.70	306.1	159.7	20	306.1	206.0	10	306.1	264.1	8
Vinclozolin	14.80	286.9	214.0	15	241.1	184.1	10	285.0	212.0	15
